# Charting Proficiency: The Learning Curve in Robotic Hysterectomy for Large Uteri Exceeding 1000 g

**DOI:** 10.3390/jcm13154347

**Published:** 2024-07-25

**Authors:** Jihyun Lee, Seongmin Kim

**Affiliations:** Gynecologic Cancer Center, CHA Ilsan Medical Center, College of Medicine, CHA University, 1205 Jungang-ro, Ilsandong-gu, Goyang-si 10414, Republic of Korea

**Keywords:** robotic hysterectomy, learning curve, large uteri, surgical proficiency, minimally invasive surgery

## Abstract

**Background/Objectives:** This study evaluates the safety and surgical outcomes of performing robotic hysterectomy on uteri weighing over 1000 g, with a focus on the surgeon’s learning curve. **Methods:** A retrospective analysis was conducted on 44 patients who underwent hysterectomy by a single surgeon from January 2020 to February 2024 using the DaVinci Xi System. Surgical procedures included total hysterectomy with bilateral salpingectomy, and specimens were removed via transvaginal manual morcellation. Operative times were segmented into docking, console, morcellation, and conversion times. **Results:** Results indicated an inflection point in the 20th case, suggesting proficiency after 20 surgeries. Comparison between early (Group A, cases 1–20) and later cases (Group B, cases 21–44) showed significant reductions in console time (CT) and morcellation time (MT) in Group B, leading to a shorter overall operative time (OT). Although estimated blood loss was higher in Group A, it was not statistically significant. Hemoglobin differences were significantly higher in Group B. No significant differences were observed in transfusion rates, postoperative analgesic usage, or complications between the groups. **Conclusions:** The study concludes that robotic hysterectomy for large uteri is safe and that surgical proficiency improves significantly after 20 cases, enhancing overall outcomes.

## 1. Introduction

Benign uterine conditions, such as uterine fibroids, endometriosis, and adenomyosis, have significant prevalence rates globally and can greatly impact women’s health [1]. The average normal uterine weight is approximately 60–80 g. However, these conditions often cause the uterus to become enlarged. Hysterectomy is one of the most commonly performed surgeries for patients with various gynecologic conditions, including uterine fibroids, adenomyosis, and endometrial hyperplasia [2]. The procedure can be performed via laparotomy or minimally invasive surgery, with minimally invasive surgery being considered the standard treatment in most cases [3]. However, open surgery may be necessary in cases of severe anatomical abnormalities, a history of complex abdominal surgeries, or when the size of the uterus limits the use of laparoscopic instruments and techniques [4].

Since the introduction of robotic surgery in 1995, its application has expanded significantly, allowing for more complex and high-difficulty surgeries to be performed minimally invasively [5]. The development of robotic surgery began in the late 20th century. Key milestones include the introduction of the DaVinci Surgical System, approved by the FDA in 2000 [6]. The DaVinci Surgical System, developed by Intuitive Surgical, revolutionized surgery by providing surgeons with enhanced capabilities, including high-definition 3D vision, wristed instruments that bend and rotate far more than the human hand, and an ergonomic console that allows surgeons to operate while seated. Over the years, advancements in robotic technology have expanded its applications, making it a valuable tool in various surgical disciplines, including gynecology. In 2014, the fourth generation Da Vinci Xi was introduced. This model offered enhanced arm mobility and flexibility, enabling access to multiple quadrants of the body without needing to reposition the patient [7]. The use of robotic instruments with superior visibility and articulating capabilities has enabled the success of these procedures. Nevertheless, performing a hysterectomy on a massive uterus using robotic surgery remains challenging due to the limited camera view, difficulties in accessing the deep pelvis, challenges in extracting the specimen, and prolonged operative times [8].

This study aims to evaluate the safety of performing a hysterectomy on uteri weighing over 1000 g using a robotic surgery system and to assess how surgical outcomes vary with the surgeon’s proficiency.

## 2. Materials and Methods

### 2.1. Study Population

This study included patients who underwent robotic-assisted hysterectomy performed by a single surgeon at a high-volume gynecologic center from January 2020 to February 2024. The surgeon had performed over 100 robotic hysterectomies and had 4 years of experience with the procedure prior to the study period. Inclusion criteria were uteri > 1000 g undergoing robotic hysterectomy. Exclusion criteria included patients with concurrent malignancies requiring additional procedures. The study was approved by the Institutional Review Board of CHA University Ilsan Medical Center (2023-03-003). Due to the retrospective nature of the study, direct consent from patients was not required.

### 2.2. Surgical Procedure

The DaVinci Xi System (Intuitive Surgical, Sunnyvale, CA, USA) was used, and the surgeon determined the number and placement of trocars for each case, using between three and five trocars. Instruments employed in the surgery included fenestrated bipolar forceps, monopolar scissors, vessel sealer extend, prograsp forceps, and a mega needle driver. A total hysterectomy with simultaneous bilateral salpingectomy was performed, and oophorectomy was optionally conducted at the discretion of the surgeon or upon the patient’s request. No additional laparotomy was performed for specimen removal; instead, the specimen was placed in a surgical bag and removed via transvaginal manual morcellation. The morcellation process was standardized as follows: First, the uterine cervix was located and clamped using a tenaculum. To secure a clear view, a narrow Deaver retractor was placed on the anterior wall of the vagina, and a duck-jaw weighted vaginal speculum was positioned on the posterior wall. Then, manual morcellation was performed using a long-handle knife. After morcellation, vaginal cuff closure was performed using intracorporeal suturing method, followed by laparoscopic lavage and closure of the abdominal incisions.

### 2.3. Definitions of Terms

For the analysis of operative times, several intervals were defined. Docking time (DT) was defined as the time taken to dock the robot to the patient. Console time (CT) encompassed the time from the surgeon sitting at the console to perform uterine resection and intracorporeal suturing. Morcellation time (MT) refers to the time spent on transvaginal morcellation, and conversion time (CVT) is the time for laparoscopic conversion, irrigation, and abdominal wound closure. The total operation time (OT) was the sum of DT, CT, MT, and CVT. Analysis was performed based on these defined intervals.

### 2.4. Statistical Analysis

Learning curves were evaluated using consecutive cases analyzed with the cumulative sum (CUSUM) method. This quantitative approach assessed the learning curves for console time, morcellation time, and total operation time (CUSUM-CT, CUSUM-MT, and CUSUM-OT, respectively). The CUSUM method provides graphical information on the trends in outcomes across consecutive procedures, plotting the cumulative differences between each data point and the overall mean. This technique visually represents the learning curve.

Statistical analysis was performed using the Statistical Package for the Social Sciences 25.0 (SPSS Inc., Chicago, IL, USA). The Kolmogorov–Smirnov test was applied to verify normal distribution assumptions. For parametric variables, Student’s *t*-test was used, while the Mann–Whitney U test was employed for non-parametric variables. Differences between proportions were compared using Fisher’s exact test or the χ^2^ test. A *p*-value of less than 0.05 was considered statistically significant.

## 3. Results

### 3.1. Patient Characteristics

**Figure 1:**
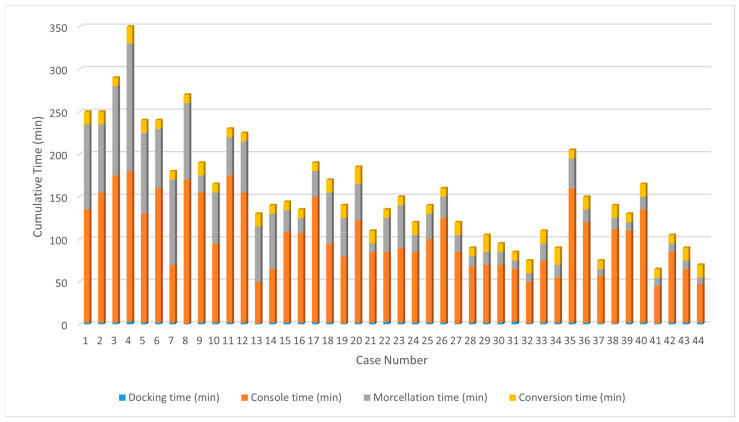
Cumulative operative times of each case, showing a decreasing trend in more recent cases.

A total of 44 patients were included in the study period. The baseline characteristics are shown in Table 1. Most patients were in their 40s and multiparous. Approximately 40% had a history of prior abdominal surgery, and the most common surgical indication was uterine fibroids. Figure 1 illustrates the overall operative times by case order, showing a decrease in OT in more recent cases.

### 3.2. Learning Curve Analysis

**Figure 2:**
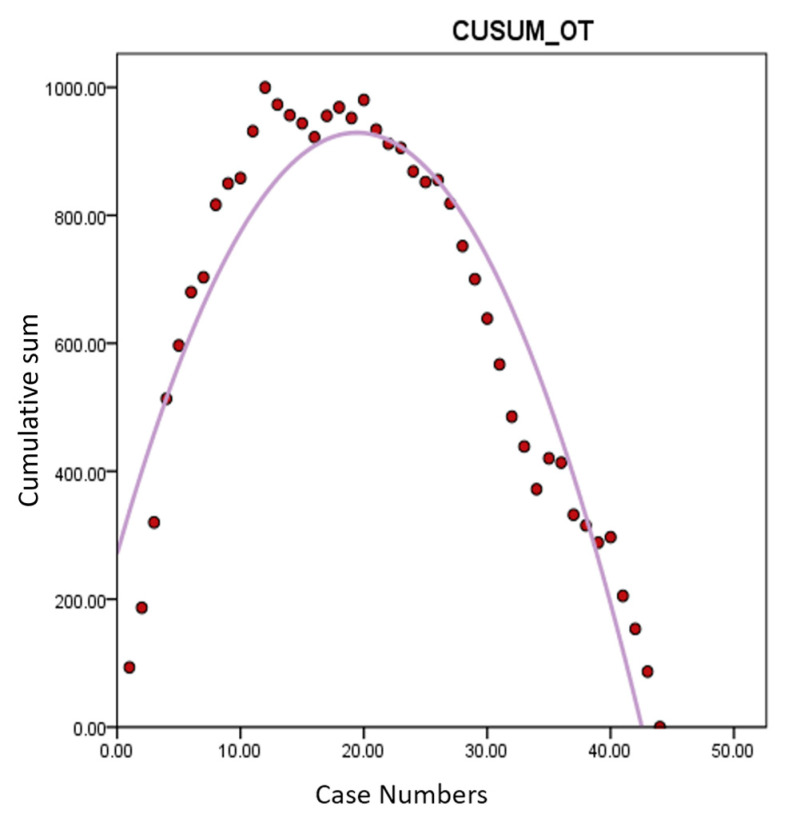
Learning curve analysis of CUSUM-OT. *Y*-axis represents the cumulative sum of total operation time. An inflection point around the 20th case indicates proficiency after approximately 20 cases of robotic hysterectomy for large uteri > 1000 g. CUSUM-OT: cumulative sum of total operation time.

**Figure 3:**
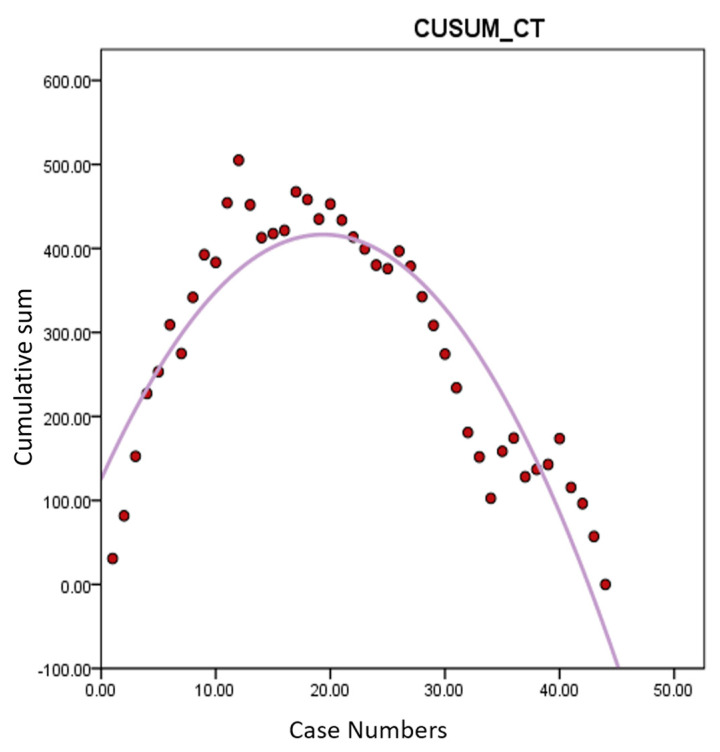
Learning curve analysis of CUSUM-CT. *Y*-axis represents the cumulative sum of console time. Similarly, a change in slope is observed around 20th case. CUSUM-CT: cumulative sum of console time.

**Figure 4:**
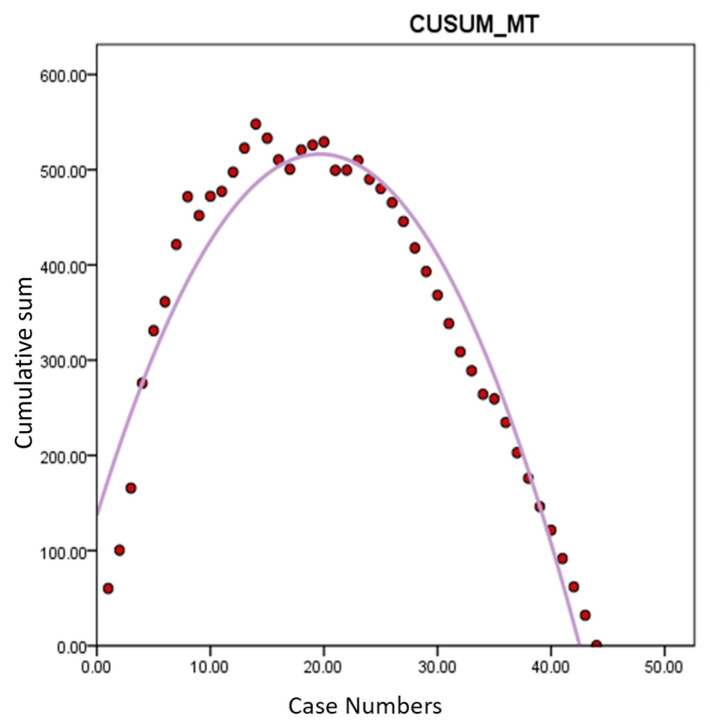
Learning curve analysis of CUSUM-MT. *Y*-axis represents the cumulative sum of morcellation time. Morcellation time also showed proficiency after 20th cases. CUSUM-MT: cumulative sum of morcellation time.

Learning curve analysis based on OT revealed an inflection point around the 20th case, indicating proficiency after approximately 20 cases (Figure 2). Similar trends were observed in the analyses of CUSUM-CT and CUSUM-MT, with inflection points around 20 cases, indicating the stabilization of operative times thereafter (Figure 3 and Figure 4). Based on this, cases were divided into Group A (cases 1–20) and Group B (cases 21–44) for comparison of surgical outcomes.

### 3.3. Comparison of Surgical Outcomes

The comparison of surgical outcomes between the two groups is detailed in Table 2. BMI and uterine weights showed no significant difference between the groups. DT and CVT also showed no significant differences. However, CT was significantly reduced in Group B (*p*-value < 0.001), and MT was notably shorter in Group B (*p*-value < 0.001), leading to a significantly longer OT in Group A (*p*-value < 0.001). Estimated blood loss was higher in Group A but not statistically significant (*p*-value = 0.057). The difference in hemoglobin levels preoperatively and postoperatively was significantly higher in Group A (*p*-value = 0.013). There were no significant differences in transfusion rates or postoperative analgesic usage. The incidence of postoperative fever over 37.5 °C was similar between the groups. Complications included wound issues in two patients and one case of cuff dehiscence in Group A, with no statistically significant differences between the groups.

## 4. Discussion

To our knowledge, this is the first study assessing the learning curve of performing robotic hysterectomy on uteri weighing over 1000 g and examining the impact of surgeon proficiency on surgical outcomes. The findings provide valuable insights into the learning curve associated with robotic hysterectomy for large uteri, highlighting the challenges and improvements that occur as the surgeon gains experience.

A previous study comparing outcomes of robotic hysterectomy versus laparotomy for uteri weighing over 1000 g found that although robotic surgery took longer (median 255 vs. 150 min, *p*-value < 0.001), it resulted in significantly less blood loss (median 150 mL vs. 425 mL) and shorter hospital stays (median 1 day vs. 2.5 days, *p*-value < 0.01) [9]. Another study examining the outcome of robotic hysterectomy versus laparoscopic hysterectomy for large uteri (≥16 weeks) found that while operative times increased with uterine weight, there were no significant differences in postoperative outcomes such as length of hospital stay or direct costs [10]. Specifically, the study showed that patients with heavier uteri experienced longer operative times and higher estimated blood loss, but overall outcomes remained comparable across different weight groups. An interesting study compared the outcomes of robotic hysterectomy and conventional laparoscopic hysterectomy by dividing the uterine weights into different categories revealed that RAH had shorter operative times and significantly lower estimated blood loss compared to TLH, particularly for uteri weighing ≥250 g [11]. The study concluded that robotic surgery offers advantages such as reduced operative time and blood loss, making it a feasible option for managing large uteri. However, despite these study results, minimally invasive surgery for large uteri remains challenging for less experienced surgeons due to the longer operative times and higher difficulty compared to open surgery.

The current analysis demonstrated that proficiency in performing robotic hysterectomy for large uteri is achieved after approximately 20 cases. This is evidenced by the significant reduction in console time (CT) and morcellation time (MT) observed after the 20th case, leading to a shorter overall operative time (OT). The learning curve, illustrated through CUSUM analysis, shows an inflection point around the 20th case, indicating a period of skill acquisition followed by stabilization and increased efficiency in surgical performance. We did not analyze the learning curve for DT and CVT. Firstly, the docking procedure of the da Vinci Xi system is relatively straightforward, allowing the surgeon to easily achieve proficiency. Given that the surgeon likely had already mastered the docking process before attempting surgeries on large uteri, the learning curve analysis for DT may not hold significant value. Additionally, CVT procedures, such as irrigation and wound closure, do not require exceptional surgical technique and were, therefore, excluded from the learning curve analysis, too. The study’s results indicate that the overall improvement in surgical speed after approximately 20 cases is due to a combination of enhanced robotic skills during console time and improved transvaginal morcellation speed for large uteri, leading to a significant reduction in total operative time [12].

The reduction in CT and MT in Group B (cases 21–44) suggests that with increased experience, surgeons become more adept at handling the complexities associated with large uteri, such as limited access to the deep pelvis and challenges in specimen extraction. This improved efficiency is crucial in minimizing operative time and potentially reducing the risks associated with prolonged surgery [13]. Despite the larger estimated blood loss in Group A (cases 1–20), the difference was not statistically significant, indicating that while repeated experience reduces blood loss, the variation between groups was not substantial enough to reach statistical significance. However, the significant difference in hemoglobin levels preoperatively and postoperatively between the groups suggests that surgical proficiency does have a positive impact on minimizing intraoperative blood loss and improving patient outcomes [14]. The estimated blood loss of the study population might be considered high for robotic hysterectomy. The higher estimated blood loss in both groups can be attributed to the larger uterine size.

The absence of significant differences in transfusion rates, postoperative analgesic usage, and complications between the two groups underscores that the safety profile of robotic hysterectomy for large uteri remains consistent, regardless of the surgeon’s experience level. This consistency is encouraging, as it suggests that even during the learning phase, the procedure can be performed safely without a notable increase in adverse outcomes [15]. In other words, although surgeries may take longer during the learning phase, which can increase the surgeon’s fatigue, the overall surgical outcomes still reflect the advantages of robotic surgery. Once proficiency is achieved, the surgery time decreases, mitigating earlier issues and maximizing the benefits of robotic surgery. This is a crucial consideration for surgeons starting with robotic procedures for large uteri.

The study’s strengths include its focus on a high-volume gynecologic center and the comprehensive analysis of operative times using the CUSUM method. However, limitations such as the retrospective design should be acknowledged. Another limitation of this study is that, aside from uterine weight, factors such as the size, volume, and shape of the uterus, which can also influence surgical difficulty, were not considered. For instance, if a large fibroid is located in the lower uterine body, it can complicate the identification and dissection of the bladder, ureter, and uterine artery, potentially affecting surgery time [16]. However, due to the retrospective nature of the study, it was challenging to account for and compare all these factors, which could impact the results. The other limitation is that the CT might include not only the time the surgeon operates the robot at the console but periods when the surgeon is not actively performing surgery as well. Since the surgeon operating at the console is not in a sterile environment, they are allowed to perform other tasks during the surgery. Additionally, if troubleshooting occurs, they must stop console operations and wait until the issue is resolved. These activities are not reflected in the data, potentially affecting the accuracy of recorded surgical times. Fortunately, Intuitive Surgical’s My Intuitive application provides data on ‘active console time’, which measures only the actual time the console is being operated [17]. Utilizing these data in research could lead to more accurate analyses of surgical times. Additionally, future studies will include a control group of uteri < 1000 g to provide baseline comparisons for operative times and outcomes.

Another important consideration is that during the study period, the surgeon performed various other surgeries in addition to the one under investigation. In clinical practice, it is rare for a surgeon to focus solely on one type of procedure continuously. As such, exploring a pure learning curve for a single technique over a period is nearly impossible [18]. Furthermore, the intervals between cases were not consistent, potentially affecting the time needed for skill acquisition [19]. However, the continuous and simultaneous development of multiple surgical techniques can positively influence each other. The progress achieved in one type of surgery can be applied to others, enhancing overall surgical proficiency. Ultimately, all surgical skills require time to master, and consistent education and training enable surgeons to continuously improve.

## 5. Conclusions

Robotic hysterectomy for uteri > 1000 g is both safe and effective. Surgical outcomes improve significantly after approximately 20 cases, highlighting the importance of experience in achieving proficiency. Additionally, based on the results of the study, it was confirmed that robotic hysterectomy for large uteri remains safe and feasible even during the learning phase. Future prospective studies with larger samples are recommended to further validate these findings.

## Figures and Tables

**Table 1:** Patient demographics and characteristics.

Characteristics	Population (n = 44)
Age	47.18 ± 4.77
BMI (kg/m^2^)	25.43 ± 4.74
Parity	
Nulliparous	2 (4.50)
Multiparous	42 (95.50)
History of previous abdominal surgery	
None	27 (61.40)
C/S only	13 (29.55)
Other MIS	3 (6.18)
Other open surgery	1 (2.27)
Main indication for hysterectomy	
Myoma	33 (75.00)
Adenomyosis	8 (18.20)
Endometrial Hyperplasia	1 (2.27)
Malignancy	2 (4.50)

Variables were shown as mean ± standard deviation or n (%). BMI; body mass index, C/S; Cesarean section, MIS; minimally invasive surgery.

**Table 2:** Surgical outcomes of study population regarding learning curve. Comparison between early (Group A, cases 1–20) and later cases (Group B, cases 21–44) showed significant reductions in console time and morcellation time in Group B, leading to a shorter overall operative time.

Surgical Outcomes	Group A (n = 20)	Group B (n = 24)	*p*-Value
BMI (kg/m^2^)	25.5 ± 4.80	25.4 ± 4.69	0.891
Uterine weight (g)	1268.05 ± 432.14	1309.54 ± 328.45	0.719
Docking time (min)	1.90 ± 0.45	1.88 ± 0.54	0.869
Console time (min)	124.8 ± 40.41	83.29 ± 29.52	<0.001
Morcellation time (min)	66.25 ± 33.66	17.75 ± 10.94	<0.001
Conversion time (min)	12.75 ± 3.43	12.92 ± 3.27	0.871
Total operation time (min)	205.7 ± 59.38	115.83 ± 34.97	<0.001
Estimated blood loss (ml)	310.00 ± 172.14	216.67 ± 144.21	0.057
Hb difference (g/dL)	1.68 ± 0.79	1.13 ± 0.60	0.013
Transfusion	2 (10.00)	0 (0.00)	0.221
PK0 (times)	0.50 ± 0.61	0.5 ± 0.59	1
PK1 (times)	0.35 ± 0.49	0.33 ± 0.48	0.91
Febrile event (≥37.5 °C)	5 (25.00)	7 (29.17)	0.757
Complications			0.086
Bladder damage	0 (0.00)	0 (0.00)	
Ureter injury	0 (0.00)	0 (0.00)	
Bowel injury	0 (0.00)	0 (0.00)	
Thromboembolic event	0 (0.00)	0 (0.00)	
Wound problem	2 (10.00)	0 (0.00)	
Cuff dehiscence	1 (5.00)	0 (0.00)	

Variables were shown as mean ± standard deviation or n (%).BMI, body mass index; Hb, hemoglobin, PK0; number of pain killer injections during the day of surgery; PK1, number of pain killer injections during the 1st postoperative day.

## Data Availability

The original contributions presented in the study are included in the article; further inquiries can be directed to the corresponding author.
